# Emerging Trends and Hot Spots in Tauopathy Research (2003–2025): A Bibliometric Analysis

**DOI:** 10.1002/brb3.71645

**Published:** 2026-08-11

**Authors:** Si‐Yao Zhang, Hui‐Jie Ren, Hao‐Yu Liu, Shu‐Ying Xu, Yong‐Jun Peng

**Affiliations:** ^1^ Department of Acupuncture and Rehabilitation Affiliated Hospital of Nanjing University of Chinese Medicine Nanjing Jiangsu Province China; ^2^ Department of Treatment of Disease Kunshan Affiliated Hospital of Nanjing University of Chinese Medicine Kunshan Jiangsu Province China; ^3^ Department of Traditional Chinese Medicine Affiliated Kunshan Hospital of Jiangsu University Kunshan Jiangsu Province China; ^4^ Department of Acupuncture‐Moxibustion, Tuina and Rehabilitation Kunshan Affiliated Hospital of Nanjing University of Chinese Medicine Kunshan Jiangsu Province China

**Keywords:** Alzheimer's disease, bibliometrics, emerging trends, hotspots, tauopathy

## Abstract

**Purpose:**

This study aimed to systematically analyze the developmental trajectory, research landscape, and thematic evolution of tauopathy research from 2003 to 2025 using bibliometric methods.

**Methods:**

A total of 6752 publications retrieved from the Web of Science Core Collection (WoSCC) were analyzed using the R package bibliometrix and VOSviewer to construct bibliometric networks, including co‐authorship, co‐citation, and keyword co‐occurrence analyses. Scimago Graphica was used to visualize international collaboration patterns.

**Results:**

The annual number of publications on tauopathy increased markedly during the period 2003–2025, reflecting sustained global research interest. The United States was the leading contributor in both publication output and citation impact, while the University of Pennsylvania emerged as a key institution. Among individual researchers, the most productive authors and influential scholars were identified through publication output and citation‐based metrics. Citation analysis highlighted the enduring impact of highly cited studies, particularly those establishing key biomarker frameworks for Alzheimer’s disease research.Keyword‐based analysis further revealed a shift from traditional mechanistic studies toward more integrative research themes, such as tau–amyloid‐β interactions, neurodegeneration, and glial involvement, alongside continued interest in tau‐targeted therapeutic strategies.

**Conclusion:**

This bibliometric analysis provides a quantitative and structured overview of the global tauopathy research landscape. Beyond confirming established patterns, it captures the dynamic evolution of research priorities and identifies emerging directions, offering a data‐driven basis for future investigations.

## Graphical abstract

This bibliometric analysis systematically evaluated global research trends in tauopathy from 2003 to 2025. Using bibliometric methods, including R package bibliometrix, VOSviewer, and Scimago Graphica, we identified publication trends, influential countries, institutions, authors, journals, and emerging research themes. The findings revealed rapid growth in tauopathy research, with increasing attention toward tau‐related mechanisms, biomarker development, neurodegeneration, and therapeutic strategies.

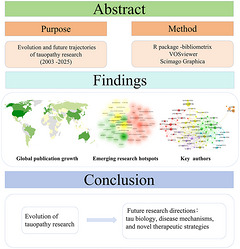

## 1 Introduction

Tau, first identified in 1975, is a member of the microtubule‐associated protein family and plays a fundamental role in regulating microtubule assembly, stability, and axonal transport in neurons (Weingarten et al. 1975). Alternative splicing of the microtubule‐associated protein tau (MAPT) gene generates six major isoforms, including three‐repeat (3R) and four‐repeat (4R) tau, which differ in their microtubule‐binding properties and biological functions. Although predominantly expressed in neurons, tau is also present in glial cells, where it participates in maintaining cytoskeletal integrity and neuronal homeostasis.

The abnormal hyperphosphorylation, misfolding, and aggregation of tau into paired helical filaments (PHFs) and neurofibrillary tangles (NFTs) represent the pathological hallmark of a heterogeneous group of neurodegenerative disorders collectively referred to as tauopathies (Lee et al. 2001). This disease spectrum includes Alzheimer's disease (AD), progressive supranuclear palsy (PSP), corticobasal degeneration (CBD), Pick's disease, and frontotemporal dementia with parkinsonism linked to chromosome 17 (FTDP‐17), all of which exhibit distinct clinical manifestations, pathological characteristics, and tau isoform compositions. The discovery of pathogenic MAPT mutations further established tau dysfunction as a primary driver of neurodegeneration rather than merely a downstream pathological consequence (Hutton et al. 1998).

**FIGURE 1:**
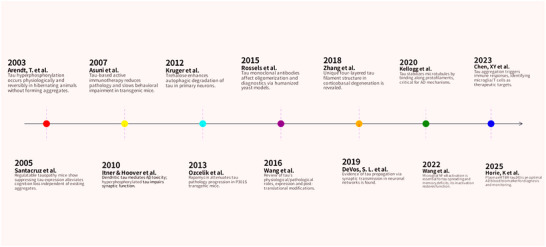
Major milestones in tauopathy research from 2003 to 2025. The timeline summarizes representative discoveries and advances that have shaped the development of tauopathy research over the past two decades.

**FIGURE 2:**
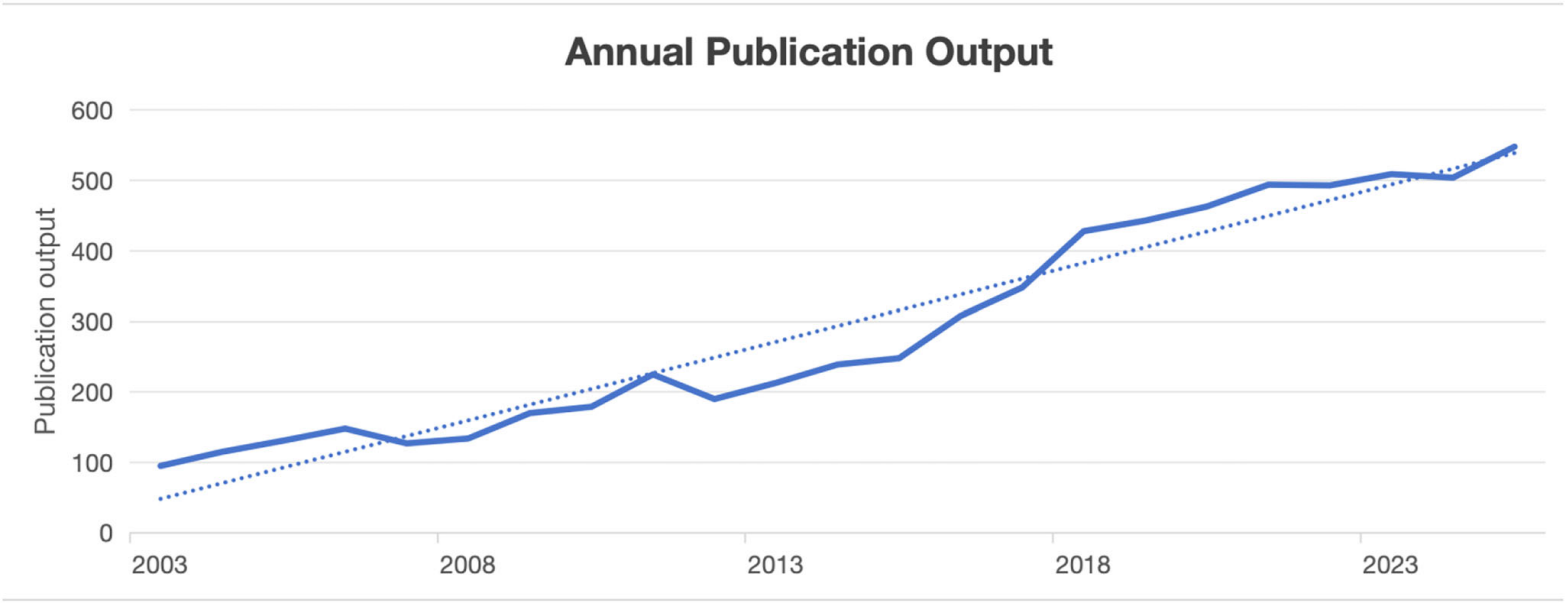
Annual publication output in tauopathy research from 2003 to 2025. Publications were retrieved from the Web of Science Core Collection (WoSCC). The solid line represents the annual publication output, and the dotted line indicates the overall publication trend.

Over the past two decades, remarkable advances in molecular biology, structural neuroscience, neuroimaging, and therapeutic development have substantially expanded our understanding of tauopathies. Breakthroughs in cryo‐electron microscopy have enabled high‐resolution characterization of disease‐specific tau filament structures, while the development of tau positron emission tomography (PET) imaging and plasma phosphorylated tau biomarkers has greatly improved the early detection and monitoring of tau‐related diseases. At the same time, increasing attention has been directed toward neuroinflammation, tau propagation, proteostasis, autophagy, and anti‐tau immunotherapies, collectively reshaping the research landscape of tauopathies. As illustrated in Figure 1, the timeline summarizes major milestones in tauopathy research over the past two decades, whereas Figure 2 demonstrates the sustained increase in annual publications, reflecting the rapid expansion of scientific interest in this field.

Bibliometric analysis has become an effective approach for quantitatively evaluating the development of scientific disciplines by integrating publication output, citation patterns, collaborative networks, and research hotspots (Chen 2004; Gao et al. 2019). In recent years, bibliometric methods have been widely applied to Alzheimer's disease and other neurodegenerative disorders, providing valuable insights into research productivity, international collaboration, and emerging scientific trends. Previous studies have investigated the overall research landscape of Alzheimer's disease as well as specific research themes, including sleep disorders and neuroinflammation, thereby identifying evolving hotspots and future research priorities (Dong et al. 2019; Tang et al. 2023; Sun et al. 2024).

Despite these valuable contributions, existing bibliometric studies have primarily focused on Alzheimer's disease as a whole or on individual pathological mechanisms rather than on tauopathy as an independent spectrum of neurodegenerative disorders. Because tauopathies encompass multiple diseases with distinct molecular mechanisms, tau isoform compositions, pathological features, and clinical phenotypes, bibliometric findings derived solely from Alzheimer's disease cannot fully capture the knowledge structure and evolutionary trajectory of the broader tauopathy field. Furthermore, the rapid emergence of fluid biomarkers, tau PET imaging, cryo‐electron microscopy, and disease‐modifying therapeutic strategies has profoundly transformed tauopathy research in recent years, highlighting the need for an updated and disease‐specific bibliometric evaluation.

Therefore, the present study conducted a comprehensive bibliometric analysis of global tauopathy research published between 2003 and 2025 using the Web of Science Core Collection (WoSCC) database. By integrating Bibliometrix, VOSviewer, and Scimago Graphica, we systematically characterized publication trends, collaborative networks, influential authors, institutions, countries, journals, and knowledge structures through co‐citation and keyword analyses. More importantly, this study identified emerging research hotspots, including neuroinflammation, fluid biomarkers, tau PET imaging, autophagy, and disease‐modifying therapeutic strategies, and evaluated their temporal evolution. Compared with previous bibliometric studies focusing on Alzheimer's disease or specific research topics, our study provides a comprehensive and up‐to‐date overview of the global tauopathy research landscape, offering valuable insights into the evolution of scientific knowledge and helping to guide future basic, translational, and clinical research.

## 2 Methods

### 2.1 Data Source and Search Strategy

The WoSCC was selected as the primary data source because it provides standardized bibliographic records, comprehensive citation information, and broad coverage of high‐quality scientific journals. Owing to its rigorous indexing standards and compatibility with widely used bibliometric software, including Bibliometrix, VOSviewer, and Scimago Graphica, WoSCC has become one of the most commonly used databases for bibliometric research (Thelwall 2008). To ensure data consistency and avoid discrepancies caused by continuous database updates, all records were retrieved and downloaded on May 16, 2026.

Relevant publications were identified using a topic‐based search strategy that combined disease‐specific terminology with tau‐related molecular keywords to maximize retrieval sensitivity while maintaining disease specificity. The search query was defined as follows: TS = (tauopathy OR tauopathies OR “progressive supranuclear palsy” OR “corticobasal degeneration” OR “Pick disease”) AND TS = (tau OR MAPT OR “tau protein”). This strategy was developed after preliminary testing of multiple search combinations to achieve an optimal balance between retrieval sensitivity and specificity. By incorporating both general tau‐related terms and representative tauopathy entities, the search strategy aimed to comprehensively capture publications across the tauopathy spectrum while minimizing the inclusion of irrelevant records.

The publication period was restricted to 2003–2025 to reflect the modern era of tauopathy research, during which substantial advances have been achieved in molecular pathology, genetics, biomarker discovery, neuroimaging, and disease‐modifying therapeutic strategies. Restricting the analysis to this period also provided a stable and representative dataset for longitudinal bibliometric evaluation.

To ensure consistency in the bibliometric analysis, only English‐language original articles and review articles were included. Editorials, meeting abstracts, letters, conference proceedings, corrections, and other non‐peer‐reviewed publication types were excluded. A total of 7450 records were initially retrieved. After applying the predefined eligibility criteria, 6752 publications were included in the final analysis, whereas 698 records were excluded. All eligible records were exported in plain text (*.txt) format for subsequent bibliometric analysis.

### 2.2 Bibliometric Analysis and Visualization

The bibliographic records retrieved from the WoSCC were imported into the bibliometrix package in R (version 5.4.0) for data conversion and descriptive bibliometric analysis. The analyses included annual publication trends, the geographical distribution of publications, authorship characteristics, and keyword co‐occurrence patterns, providing a comprehensive overview of the development of tauopathy research.

To evaluate the academic influence of individual authors, several bibliometric indicators were calculated, including the number of publications, total citations, the h‐index, and the m‐index. The m‐index was calculated by dividing the h‐index by the academic age. In the present study, academic age was defined as the number of years since an author's first tauopathy‐related publication within the retrieved WoSCC dataset rather than the author's first publication across all research fields (Hirsch 2005).

Network construction and visualization were performed using VOSviewer (version 1.6.20). Co‐authorship analyses were conducted at the country/region, institutional, and author levels, whereas co‐citation analyses were performed for journals and cited references. In addition, citation analysis of publications and keyword co‐occurrence analysis were carried out to identify influential studies and emerging research topics. The full counting method and the default normalization settings in VOSviewer were applied throughout all network analyses.

To further illustrate international research collaborations, Scimago Graphica was used to visualize collaboration networks among countries and regions, providing a clearer representation of global collaborative relationships.

### 2.3 Bibliometric Analysis Parameters

To ensure methodological transparency and reproducibility, threshold values were predefined for all bibliometric analyses. The predefined threshold values were determined based on the distribution of publication output, citation frequencies, and co‐citation frequencies within the retrieved dataset to achieve an appropriate balance between network density, visualization clarity, and analytical interpretability while retaining the most representative contributors and research themes in each bibliometric network.

For country‐level analysis, countries/regions were ranked according to publication output and total citation counts. The top 10 countries/regions were identified as the most productive and influential contributors. In the collaboration network analysis, only countries/regions with more than 75 publications were included, and the top 20 most productive countries/regions were visualized.

For institutional analysis, institutions were ranked according to publication output and total citation counts. Institutions with more than 73 publications were included in the co‐authorship network, and the top 30 institutions were visualized. The top 10 institutions ranked by publication output were additionally summarized in a table. The average publication year of each institution was calculated using VOSviewer to illustrate temporal trends in institutional research activity.

For journal analysis, journals were ranked according to publication output and total citation counts. The top 10 journals based on publication output and the top 10 journals based on total citation counts were identified as the most productive and the most influential publication sources, respectively.

For author analysis, authors were ranked according to publication output and total citation counts. The top 10 authors were identified as the most productive and the most influential contributors. In the co‐authorship analysis, only authors with at least 25 publications were included. Isolated nodes were excluded from the visualization to improve network readability without affecting the overall collaboration structure.

For keyword analysis, keywords were ranked according to their frequency of occurrence. Only keywords with a minimum occurrence of 65 were included in the co‐occurrence network, and the 50 most frequently occurring keywords were selected for word cloud visualization. To facilitate interpretation, keywords were further categorized into disease‐, pathology‐, and molecule‐related terms according to their semantic characteristics, and the top 10 keywords in each category were presented. In addition, the average publication year of keywords was calculated using VOSviewer to illustrate temporal changes in research topics, and temporal keyword evolution was analyzed to identify emerging research trends in tauopathy.

For citation analysis, publications with at least 500 citations were identified as highly cited studies. After confirming their relevance to tauopathy research based on their titles and abstracts, the top 10 most cited publications were selected according to total citation counts. In the co‐citation analysis, references with at least 260 co‐citations were included to identify the intellectual foundation of the field. The top 10 co‐cited references were then ranked according to co‐citation frequency.

## 3 Results

### 3.1 Distribution of Publications by Year

Between 2003 and 2025, scientific output on tauopathy increased substantially. A total of 6752 publications were included in the analysis, comprising 5638 original articles (83.5%) and 1114 review articles (16.5%). As shown in Figure 2, the annual publication output exhibited a consistent upward trend throughout the study period. The number of publications increased from 95 in 2003 to 548 in 2025, representing a 476.8% increase. Annual publication output exceeded 200 for the first time in 2011 and continued to grow steadily thereafter, rising from approximately 200 publications per year to more than 500 publications per year over the past decade. Overall, these findings demonstrate a sustained expansion of research activity on tauopathy.

### 3.2 Countries

**FIGURE 3:**
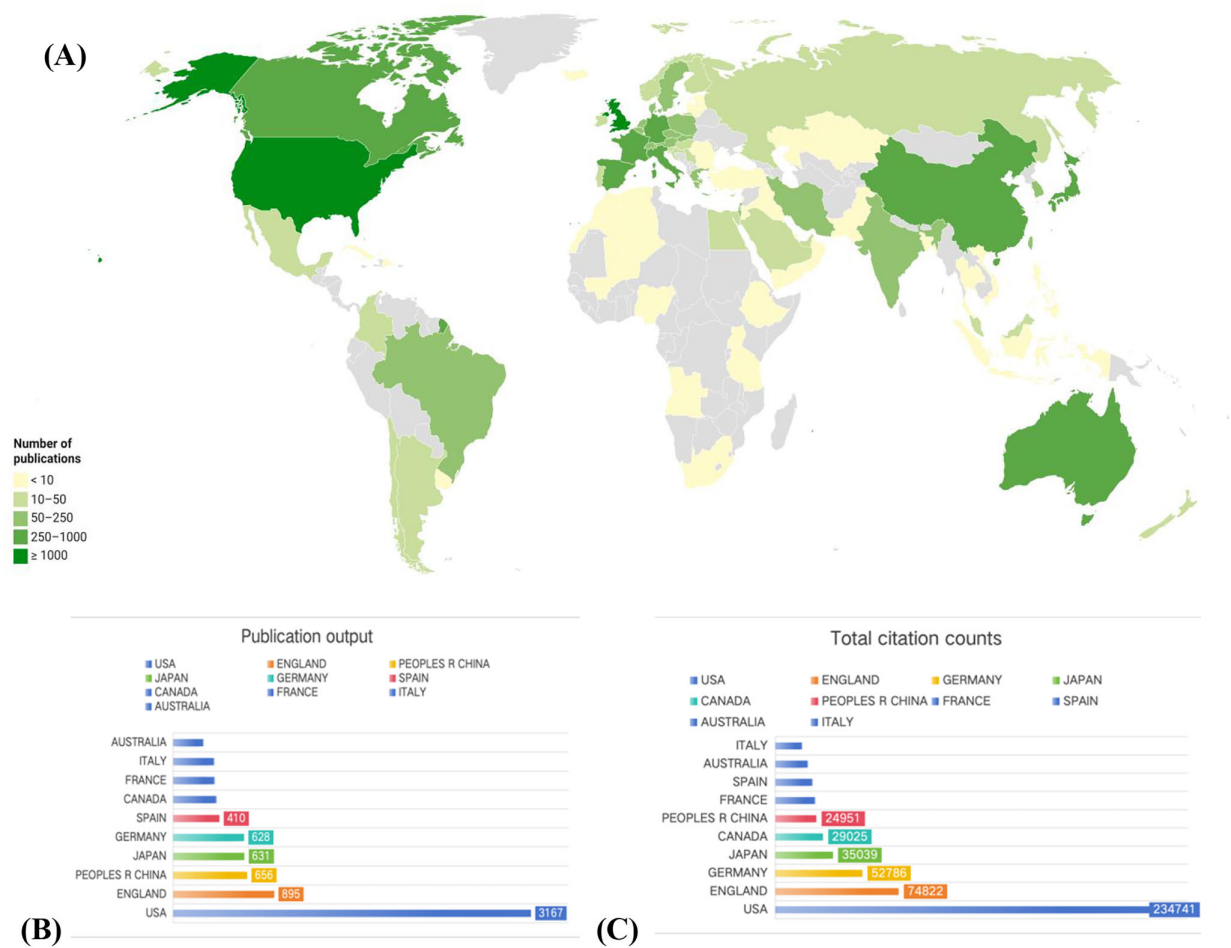
Global distribution and scientific contributions of countries/regions in tauopathy research. (A) Global distribution of publication output in tauopathy research based on publications retrieved from the Web of Science Core Collection (WoSCC). Color intensity represents publication output, whereas gray indicates no publications retrieved from the dataset. (B) Publication output of the 10 most productive countries/regions. (C) Top 10 countries/regions ranked by total citation counts, reflecting their academic impact in tauopathy research.

Figure 3A illustrates the global distribution of publication output on tauopathy, involving 94 countries and regions. The United States had the highest publication output, accounting for 46.90% of all publications, with 3167 articles. England ranked second with 895 publications (13.21%), followed by China (656, 9.72%), Japan (631, 9.35%), and Germany (628, 9.30%) (Figure 3B). Spain, Canada, France, Italy, and Australia also ranked among the top 10 countries in terms of publication output.

In terms of citation impact, the United States ranked first with 234,741 total citations, followed by England (74,822), Germany (52,786), Japan (35,039), and Canada (29,025) (Figure 3C). China, France, Spain, Australia, and Italy also ranked among the top 10 countries by total citations, indicating their important influence in tauopathy research.

**FIGURE 4:**
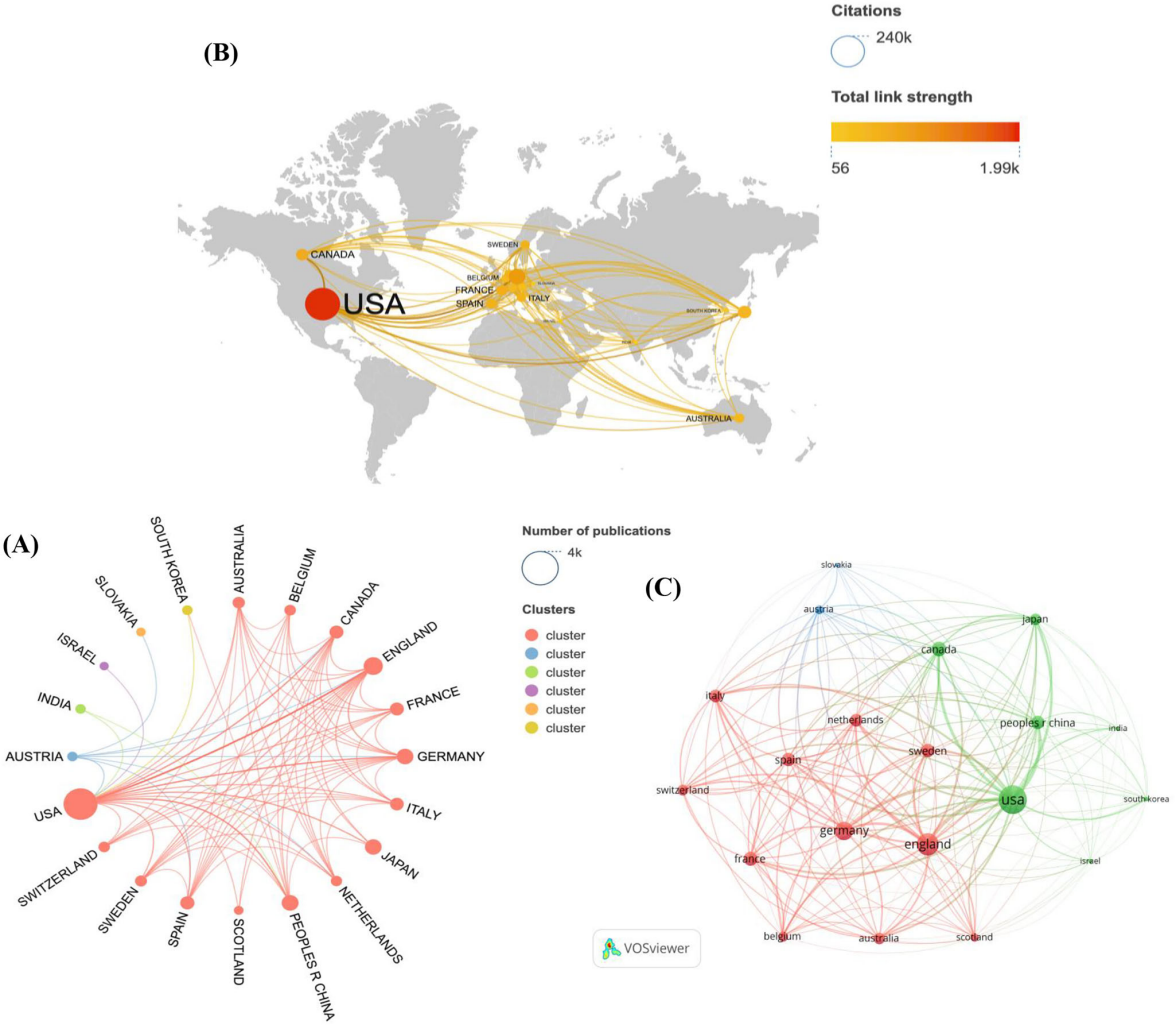
International collaboration networks among countries/regions in tauopathy research. (A) Network visualization of international collaborations among countries/regions. Node size represents publication output, link thickness indicates collaboration strength, and node colors represent different collaboration clusters identified by VOSviewer. (B) Geographic visualization of international collaboration among countries/regions. Node size corresponds to publication output, and connecting lines indicate collaborative relationships. (C) Cluster visualization of the country/region collaboration network. Different colors represent distinct collaboration clusters, and links indicate collaborative relationships between countries/regions.

Figure 4 presents the international collaboration network among countries and regions involved in tauopathy research. Collaboration was widespread, with the United States occupying the most central position in the global network. England, Germany, Canada, and France also maintained extensive collaborative relationships with other countries.

To further evaluate collaboration patterns, Figure 4C displays the 20 countries and regions with more than 75 publications. The United States had the highest total link strength (1984), followed by England (1320), Germany (936), Canada (648), and France (566). These results indicate that the United States played a leading role in promoting international collaboration in tauopathy research, while several European countries and Canada also served as important collaborative partners.

### 3.3 Organizations

A total of 5178 organizations contributed to tauopathy research during the study period. To investigate institutional collaboration, a co‐authorship analysis was performed on the 30 organizations with more than 73 publications. The top 10 organizations ranked by publication output are listed in Table 1. Mayo Clinic ranked first with 343 publications (5.08%), followed by the University of Pennsylvania (308, 4.56%), University of California, San Francisco (235, 3.48%), University College London (217, 3.21%), and the University of Cambridge (156, 2.31%). As shown in Table 1, institutions from the United States, including Mayo Clinic, the University of Pennsylvania, and the University of California, San Francisco, received the highest total citation counts. University College London and the University of Cambridge also ranked among the most highly cited institutions, reflecting their substantial academic influence in tauopathy research.

**TABLE 1:** Top 10 most productive organizations.

Rank	Organizations	Country	Publications	Citations	Total link strength
1	Mayo Clinic	USA	343	36,156	304
2	University of Pennsylvania	USA	308	37,700	278
3	University of California, San Francisco	USA	235	22,453	281
4	University College London	UK	217	20,201	232
5	University of Cambridge	UK	156	14,698	110
6	Harvard Medical School	USA	150	10,488	198
7	Washington University	USA	149	20,419	171
8	German Center for Neurodegenerative Diseases (DZNE)	Germany	147	12,902	112
9	University of Toronto	Canada	131	7973	156
10	Boston University	USA	129	16,795	147

**FIGURE 5:**
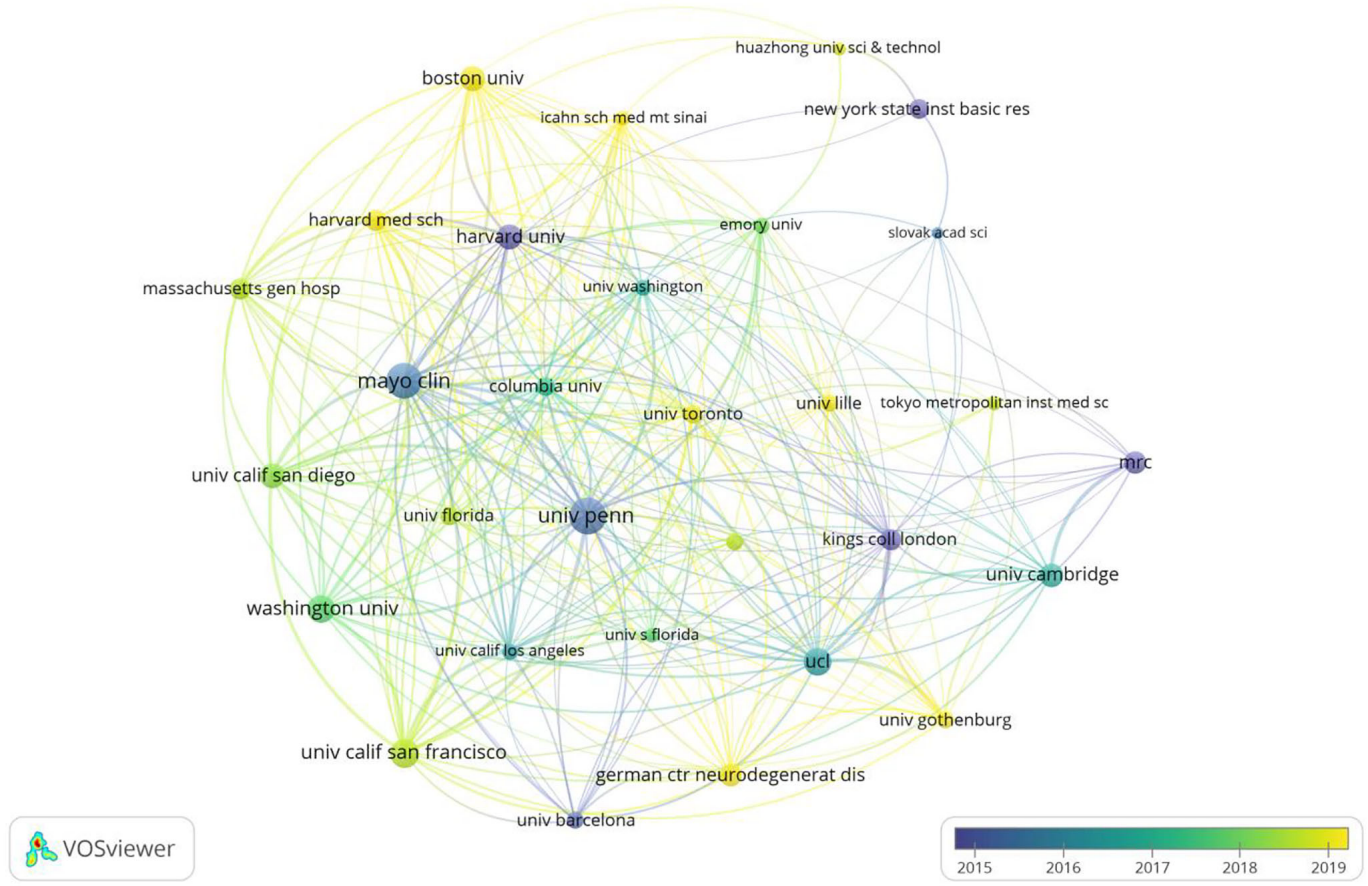
Overlay visualization of institutional collaboration in tauopathy research. Nodes represent institutions, with node size proportional to publication output. Links indicate collaborative relationships between institutions. Node colors represent the average publication year, ranging from earlier (blue) to more recent (yellow), illustrating the temporal evolution of institutional research activity.

Figure 5 illustrates the collaboration network among the selected organizations. Extensive collaborative relationships were observed among the leading institutions, particularly those in the United States and the United Kingdom. The overlay visualization further indicates the temporal distribution of institutional publication activity. Organizations shown in yellow had a more recent average publication year. Harvard Medical School and Boston University were among the institutions with relatively recent publication activity, suggesting their increasing involvement in tauopathy research. Overall, these findings demonstrate that several leading institutions have maintained high publication output, substantial citation impact, and extensive collaborative relationships in tauopathy research.

### 3.4 Journals and Cited Journals

A total of 845 journals published research on tauopathy during the study period. The top 10 journals ranked by publication output are presented in Table 2. Journal of Alzheimer's Disease ranked first with 307 publications (4.51% of all publications), followed by Acta Neuropathologica (253, 3.72%), Acta Neuropathologica Communications (186, 2.73%), Journal of Biological Chemistry (158, 2.32%), and Neurobiology of Aging (149, 2.19%). Table 2 also summarizes the citation impact of the leading journals. Acta Neuropathologica received the highest total citation count (18,547), followed by Journal of Biological Chemistry (17,133), Proceedings of the National Academy of Sciences of the United States of America (15,389), Journal of Neuroscience (13,472), and Neurology (13,204). These journals represent the major publication venues with the greatest academic influence in tauopathy research. Overall, the results demonstrate that a relatively small number of journals account for a substantial proportion of both publication output and citation impact in tauopathy research.

**TABLE 2:** Top 10 prolific journals and cited journals.

	Prolific journals				Cited journals			
Rank	Journals	Records	2025 Impact factor	2025 JCR partition	Journals	Citations	2025 Impact factor	2025 JCR partition
1	J Alzheimers Dis	307	3.1	Q2	Acta Neuropathol	18,547	9.3	Q1
2	Acta Neuropathol	253	9.3	Q1	J Biol Chem	17,133	3.9	Q2
3	Acta Neuropathol Commun	186	5.7	Q1	P Natl Acad Sci USA	15,389	9.1	Q1
4	J Biol Chem	158	3.9	Q2	J Neurosci	13,472	4.0	Q2
5	Neurobiol Aging	149	3.5	Q2	Neurology	13,204	9.0	Q1
6	Brain	132	11.7	Q1	Neuron	11,401	15.3	Q1
7	Int J Mol Sci	129	4.9	Q1	Brain	10,602	11.7	Q1
8	Neurobiol Dis	126	5.6	Q1	Nature	9511	48.5	Q1
9	J Neuropathol Exp Neurol	120	4.2	Q2	Ann Neurol	8834	7.7	Q1
10	Alzheimers Dement	115	11.1	Q1	J Alzheimers Dis	8137	3.1	Q2

### 3.5 Authors and Co‐Authorship of Authors

**FIGURE 6:**
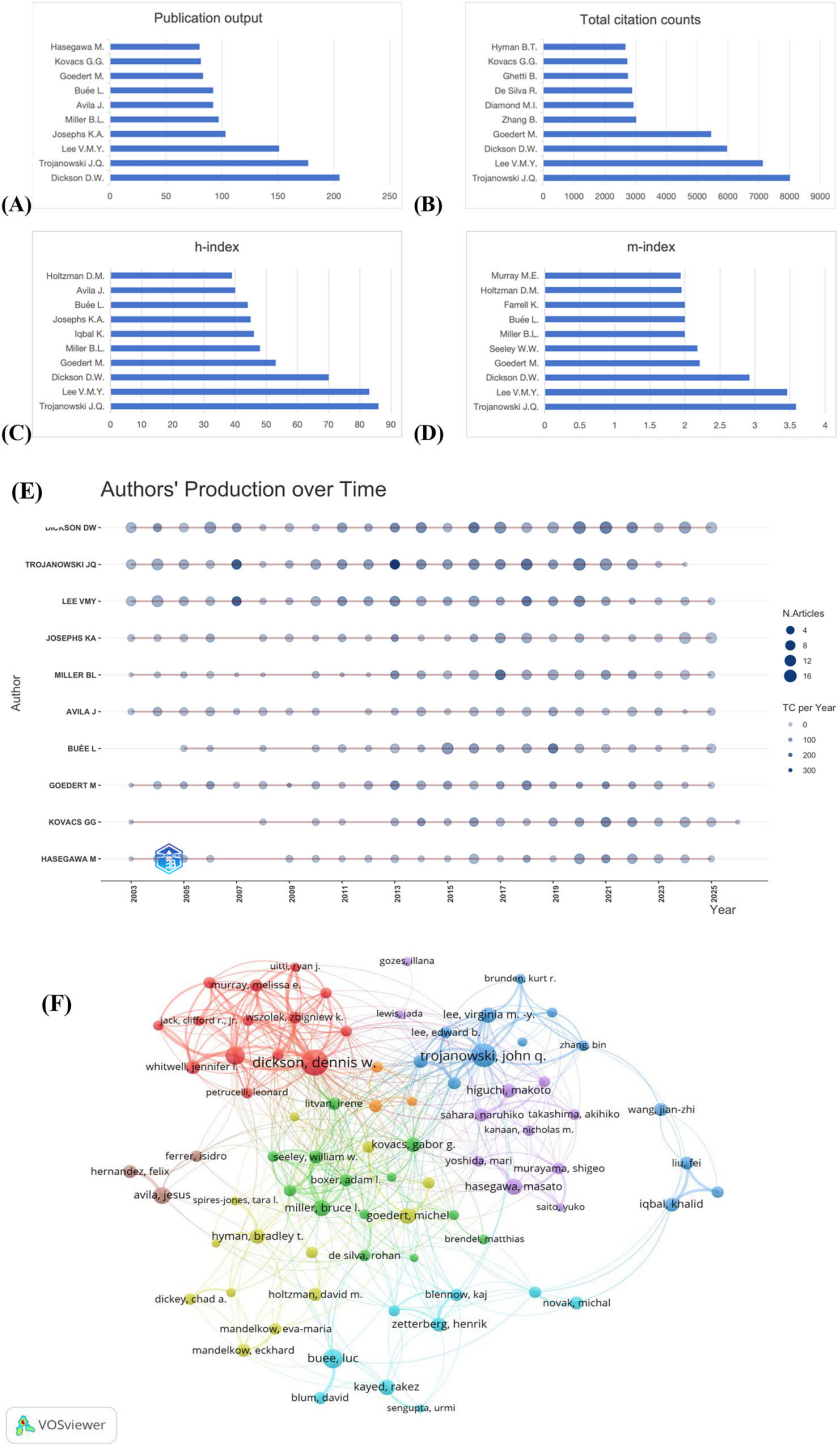
Bibliometric analysis of authors in tauopathy research. (A) Top 10 authors ranked by publication output. (B) Top 10 authors ranked by total citation counts. (C) Top 10 authors ranked by h‐index. (D) Top 10 authors ranked by m‐index. (E) Publication output of the top 10 authors over time. Bubble size represents the number of publications, and bubble color intensity represents total citations per year. (F) Network visualization of co‐authorship among authors. Node size represents publication output, links indicate co‐authorship relationships, and different colors represent distinct collaboration clusters identified by VOSviewer.

A total of 27,495 authors contributed to tauopathy research during the study period. In terms of publication output, Dickson D.W. ranked first with 205 publications, followed by Trojanowski J.Q. (177, 2.62%), Lee V.M.‐Y. (151, 2.24%), Josephs K.A. (103, 1.53%), and Miller B.L. (97, 1.44%) (Figure 6A).

Figure 6B presents the top 10 authors ranked by total citations. Trojanowski J.Q. received the highest total citations (8013), followed by Lee V.M.‐Y. (7130), Dickson D.W. (5967), Goedert M. (5454), and Zhang B. (3016). Regarding the h‐index, Trojanowski J.Q. ranked first (86), followed by Lee V.M.‐Y. (83), Dickson D.W. (70), Goedert M. (53), and Miller B.L. (48) (Figure 6C). Similarly, Trojanowski J.Q. showed the highest m‐index (3.583), followed by Lee V.M.‐Y. (3.458), Dickson D.W. (2.917), Goedert M. (2.208), and Seeley W.W. (2.176) (Figure 6D).

Figure 6E illustrates the temporal distribution of the top 10 authors with the highest publication output, demonstrating the evolution of author productivity over time. The co‐authorship network analysis included 83 authors with more than 25 publications. After excluding two isolated authors, 81 authors remained in the final network (Figure 6F). Dickson D.W. exhibited the highest total link strength (TLS = 449), followed by Trojanowski J.Q. (TLS = 295), Josephs K.A. (TLS = 279), Murray M.E. (TLS = 187), and Wszolek Z.K. (TLS = 181). These findings demonstrate the contribution of key researchers and the collaborative structure of the tauopathy research community.

### 3.6 Keywords

**FIGURE 7:**
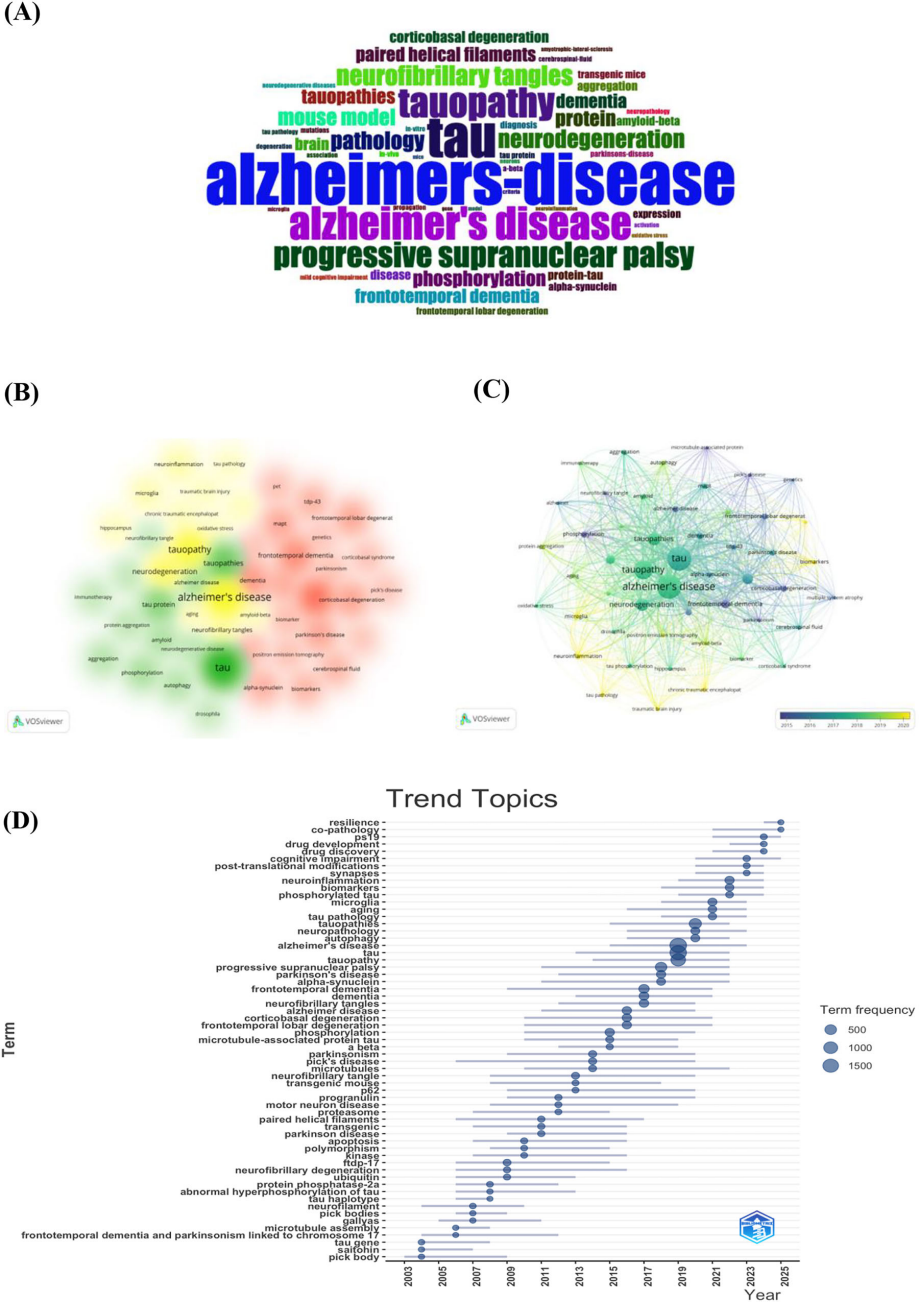
Bibliometric analysis of keywords in tauopathy research. (A) Word cloud of the 50 most frequently occurring keywords. Word size is proportional to keyword occurrence frequency. (B) Density visualization of the keyword co‐occurrence network, where darker colors indicate higher keyword occurrence density and represent major research hotspots. (C) Overlay visualization of keyword co‐occurrence. Node size represents keyword occurrence frequency, links indicate co‐occurrence relationships, and node colors represent the average publication year, ranging from earlier (blue) to more recent (yellow). (D) Temporal evolution of research topics identified by trend topics analysis. Bubble size represents term frequency, and horizontal lines indicate the time span during which each topic remained active.

A total of 8474 keywords were identified, among which 50 keywords occurred more than 65 times. Figure 7A presents the word cloud of the 50 most frequently occurring keywords. Alzheimer's disease was the most frequently occurring keyword, followed by tau, neurodegeneration, pathology, and paired helical filaments.

Figure 7B presents the keyword density visualization, in which darker colors indicate higher keyword density and lighter colors indicate lower density. Figure 7C shows the overlay visualization of keywords, with colors representing the average publication year (APY) of associated publications. Most frequently occurring keywords were distributed between 2017 and 2019, while keywords shown in yellow represented relatively recent research topics with higher APY values, including biomarkers, neuroinflammation, and tau pathology. Furthermore, the trend topics analysis in Figure 7D illustrates the temporal evolution of major research themes in tauopathy, identifying changes in research focus and emerging topics over time.

Table 3 summarizes the top 10 diseases, pathophysiology, and molecules associated with tauopathy research. Among diseases, Alzheimer's disease, PSP, and frontotemporal dementia were the most frequently occurring keywords. Regarding pathological processes, neurodegeneration, phosphorylation, and protein aggregation were among the most extensively investigated topics. In addition, amyloid‐beta, MAPT, and alpha‐synuclein were the most frequently studied molecules.

**TABLE 3:** Top 10 diseases, pathologies, and key molecules involved in tauopathy research.

Rank	Disease	Occurrence	Pathophysiology	Occurrence	Key molecules	Occurrence
1	Alzheimer's disease	2194	Neurodegeneration	613	Amyloid‐beta	347
2	Progressive supranuclear palsy	576	Phosphorylation	336	Microtubule‐associated protein tau	241
3	Frontotemporal dementia	305	Protein aggregation	276	Alpha‐synuclein	132
4	Parkinson's disease	280	Neurofibrillary tangles	264	Tdp‐43	129
5	Chronic traumatic encephalopathy	102	Neuroinflammation	228	Microtubules	111
6	Traumatic brain injury	94	Corticobasal degeneration	185	Tau oligomers	40
7	Corticobasal syndrome	79	Frontotemporal lobar degeneration	204	Paired helical filaments	32
8	Pick's disease	72	Autophagy	136	Apoe	31
9	Multiple system atrophy	67	Aging	107	Hyperphosphorylated tau	31
10	Ftdp‐17	63	Hyperphosphorylation	103	Progranulin	30

Keyword co‐occurrence and trend topic analyses revealed the evolving research landscape of tauopathy studies. Recent research attention has increasingly focused on neuroinflammation, autophagy, and biomarker‐related studies, suggesting their emergence as important research directions.

### 3.7 Citation and Co‐Citation

**FIGURE 8:**
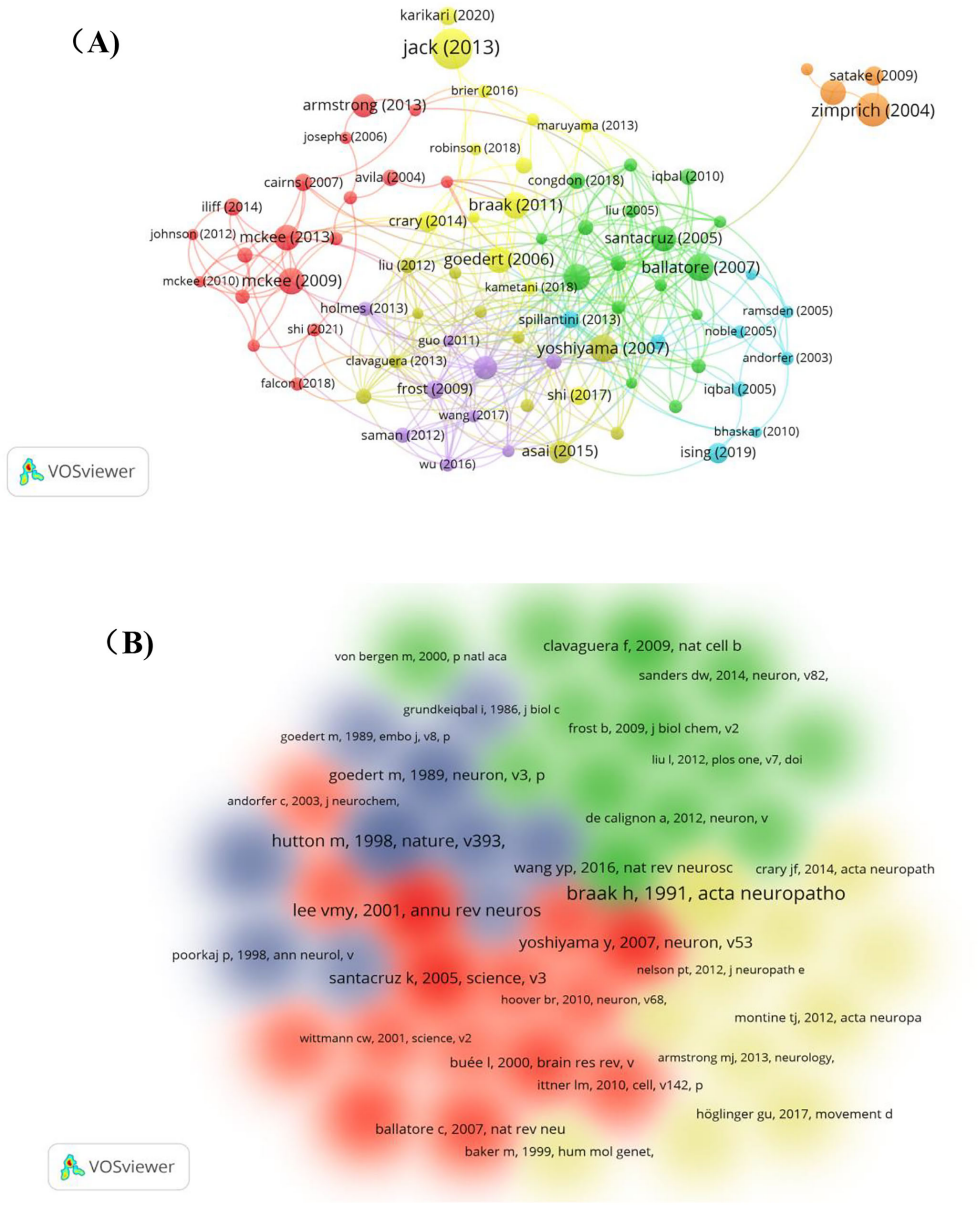
Bibliometric analysis of citation and co‐citation networks in tauopathy research. (A) Citation network of publications with at least 500 citations. Node size represents citation frequency; links indicate citation relationships between publications; and node colors represent different citation clusters. (B) Density visualization of the reference co‐citation network, including references with at least 260 co‐citations. Darker colors indicate higher co‐citation density, whereas lighter colors indicate lower co‐citation density.

Citation analysis identified 83 highly cited documents with more than 500 citations. After manual screening, two documents unrelated to tauopathy research were excluded (Figure 8A). Based on citation counts, the top 10 highly cited documents are presented in Table 4. The citation counts of these documents ranged from 1595 to 3262. The most highly cited document was “Tracking pathophysiological processes in Alzheimer's disease: an updated hypothetical model of dynamic biomarkers,” published by Jack CR in 2013 in *The Lancet Neurology*, with 3262 citations. The second‐ranked document was “Mutations in LRRK2 cause autosomal‐dominant Parkinsonism with pleomorphic pathology” by Zimprich et al. (2004), with 2427 citations, followed by “Tau‐mediated neurodegeneration in Alzheimer's disease and related disorders” by Ballatore et al. (2007), with 1825 citations.

**TABLE 4:** Top 10 highly citation analysis of publications in the field of tauopathy research.

Rank	Title	First author	Journals	Publication year	Total citations
1	Tracking pathophysiological processes in Alzheimer's disease: an updated hypothetical model of dynamic biomarkers	Jack CR	Lancet Neurol.	2013	3262
2	Mutations in LRRK2 cause autosomal‐dominant Parkinsonism with pleomorphic pathology	Zimprich A	Neuron	2004	2427
3	Tau‐mediated neurodegeneration in Alzheimer's disease and related disorders	Ballatore C	Nat. Rev. Neurosci.	2007	1825
4	Synapse loss and microglial activation precede tangles in a P301S tauopathy mouse model	Yoshiyama Y	Neuron	2007	1790
5	A century of Alzheimer's disease	Goedert M	Science	2006	1763
6	Chronic Traumatic Encephalopathy in Athletes: Progressive Tauopathy After Repetitive Head Injury	McKee AC	J. Neuropathol. Exp. Neurol.	2009	1725
7	Tau in physiology and pathology	Wang YP	Nat. Rev. Neurosci.	2016	1704
8	Stages of the Pathologic Process in Alzheimer Disease: Age Categories From 1 to 100 Years	Braak H	J. Neuropathol. Exp. Neurol.	2011	1700
9	Tau Suppression in a Neurodegenerative Mouse Model Improves Memory Function	Santacruz K	Science	2005	1655
10	Genome‐wide association study reveals genetic risk underlying Parkinson's disease	Simón‐Sánchez J	Nat. Genet.	2009	1595

A co‐citation network of cited references was constructed using a minimum citation threshold of 260, resulting in 49 highly cited references included for further analysis (Figure 8B). Table 5 presents the top 10 co‐cited references in tauopathy research. The most co‐cited reference was Braak H et al. (1991) published in *Acta Neuropathologica* (total link strength [TLS] = 5664), followed by Hutton M et al. (1998) in *Nature* (TLS = 5210), Clavaguera F et al. (2009) in *Nature Cell Biology* (TLS = 4239), Lee VMY et al. (2001) in *Annual Review of Neuroscience* (TLS = 4197), and Goedert M et al. (1989) in *Neuron* (TLS = 4034). Overall, these influential publications form the core knowledge base of tauopathy research and have profoundly influenced subsequent investigations into tau pathology, genetic mechanisms, disease progression, and neurodegeneration.

**TABLE 5:** Top 10 highly co‐citation analysis of publications in the field of tauopathy research.

Rank	Title	First author	Journals	Publication year	Co‐citations(TLS)
1	Neuropathological stageing of Alzheimer‐related changes	Braak H	Acta Neuropathol.	1991	5664
2	Association of missense and 5′‐splice‐site mutations in tau with the inherited dementia FTDP‐17	Hutton M	Nature	1998	5210
3	Transmission and spreading of tauopathy in transgenic mouse brain	Clavaguera F	Nat. Cell Biol.	2009	4239
4	Neurodegenerative tauopathies	Lee VMY	Annu. Rev. Neurosci.	2001	4197
5	Multiple isoforms of human microtubule‐associated protein tau: sequences and localization in neurofibrillary tangles of Alzheimer's disease	Goedert M	Neuron	1989	4034
6	Tau Suppression in a Neurodegenerative Mouse Model Improves Memory Function	Santacruz K	Science	2005	3871
7	Synapse loss and microglial activation precede tangles in a P301S tauopathy mouse model	Yoshiyama Y	Neuron	2007	3492
8	A protein factor essential for microtubule assembly	Weingarten MD	Proc. Natl. Acad. Sci. USA.	1975	3240
9	Mutation in the tau gene in familial multiple system tauopathy with presenile dementia	Spillantini MG	Proc. Natl. Acad. Sci. USA.	1998	3156
10	Propagation of tau misfolding from the outside to the inside of a cell	Frost B	J. Biol. Chem.	2009	3063

## 4 Discussion

### 4.1 Global Research Landscape of Tauopathy

The present bibliometric analysis provides a comprehensive overview of the global research landscape of tauopathy from 2003 to 2025, revealing a sustained increase in publication output together with the evolution of international collaboration and scientific influence. Rather than simply reflecting increased research activity, this upward trend mirrors the growing recognition of tau pathology as a central mechanism underlying multiple neurodegenerative diseases and the expanding interest in developing disease‐modifying therapeutic strategies.

Several major scientific advances have likely contributed to this rapid growth. Progress in structural biology, biomarker discovery, neuroimaging, and tau‐targeted therapeutic development has substantially improved the understanding of tau pathology while facilitating earlier diagnosis and translational research. These advances have transformed tauopathy research from a predominantly pathology‐oriented discipline into an increasingly multidisciplinary field that integrates molecular neuroscience, clinical neurology, neuroimaging, and biomarker research.

The United States remained the leading contributor in terms of publication output, citation impact, and international collaboration, highlighting its central role in shaping the development of tauopathy research. This leadership is likely supported by sustained research funding, well‐established multidisciplinary research infrastructure, and extensive collaborative networks. Leading institutions, particularly the Mayo Clinic, have made substantial contributions to the field through influential studies on tau pathology, disease mechanisms, and biomarker development. Although North America and Western Europe continue to dominate the field, increasing publication output from countries such as China and Japan reflects expanding global participation. Nevertheless, collaborative networks and citation impact remain concentrated among a limited number of countries and institutions, indicating that disparities in research capacity still exist. Strengthening international collaboration and promoting multidisciplinary research partnerships may therefore further accelerate scientific progress in tauopathy research.

Overall, the observed publication and collaboration patterns suggest that tauopathy research has entered a stage of rapid expansion characterized by increasing interdisciplinarity and translational orientation. These findings not only illustrate the global evolution of the field but also provide an important context for interpreting the thematic evolution and emerging research hotspots discussed in the following sections.

### 4.2 Comparison With Previous Bibliometric Studies

Previous bibliometric studies have provided valuable insights into the development of Alzheimer's disease (AD) and other neurodegenerative disease research by characterizing publication output, collaboration networks, influential contributors, and emerging research hotspots. Recent bibliometric studies on sleep and Alzheimer's disease, neuroinflammation in Alzheimer's disease, and global Alzheimer's disease research have consistently highlighted the increasing importance of tau pathology, biomarkers, neuroinflammation, and disease‐modifying therapeutic strategies, thereby reflecting the growing shift toward mechanism‐oriented and translational research in neurodegenerative diseases (Dong et al. 2019; Tang et al. 2023; Sun et al. 2024).

However, these studies primarily focused on Alzheimer's disease or broader neurodegenerative disorders rather than tauopathies as a distinct group of disorders. Consequently, the evolution of tau‐centered research across multiple primary tauopathies has remained insufficiently characterized, particularly regarding thematic evolution, emerging research directions, and the intellectual structure of the field.

The present study addresses these gaps by providing a comprehensive bibliometric analysis of global tauopathy research spanning the period from 2003 to 2025. By integrating co‐authorship, co‐citation, keyword co‐occurrence, and temporal keyword evolution analyses, this study not only characterizes the collaborative landscape but also reveals the dynamic transition of research priorities from classical neuropathology to biomarker development, neuroinflammation, glial biology, experimental models, and tau‐targeted therapeutic strategies.

Overall, this study complements existing bibliometric literature by providing a tauopathy‐specific and up‐to‐date perspective on the evolution of the field. By integrating publication trends with collaboration networks, citation structures, and thematic evolution, this work offers a comprehensive framework for understanding the historical development, current research priorities, and potential future directions of tauopathy research.

### 4.3 Intellectual Foundation of Tauopathy Research

Authorship and co‐citation analyses identified several influential researchers and landmark publications that have collectively shaped the intellectual foundation of tauopathy research. Their sustained academic influence reflects the evolution of the field from neuropathological characterization toward mechanistic investigation, biomarker development, and translational research.

Among the most influential contributors, Trojanowski JQ. and Lee VMY. consistently ranked among the leading authors in terms of publication output and citation impact. Their long‐term collaboration has substantially advanced the understanding of tau biology, particularly in tau aggregation, propagation, and the neuropathological classification of tau‐related neurodegenerative disorders (Kovacs et al. 2018; Xu and In 2023; Marshall et al. 2022; Gibbons et al. 2020; Henderson et al. 2021). In addition, Dickson DW. has made major contributions to the classification of primary tauopathies and experimental disease models, while Goedert M. has played a pivotal role in elucidating tau filament structure and the molecular mechanisms underlying tau aggregation (Lee et al. 2001; Lewis et al. 2000; Estades Ayuso et al. 2023; Shi et al. 2021). Together, these investigators have established much of the conceptual framework that underpins contemporary tauopathy research.

Co‐citation analysis further demonstrated that several landmark publications continue to exert substantial influence on the field. The biomarker framework proposed by Jack and colleagues remains one of the most highly co‐cited references, reflecting the transition from symptom‐based diagnosis to biomarker‐based disease classification in Alzheimer's disease (Jack et al. 2013, 2010, 2018). Likewise, the seminal work by (Braak and Braak, 1991) describing the hierarchical progression of neurofibrillary pathology continues to serve as a cornerstone for understanding tau pathology and disease progression.

Collectively, these findings indicate that the intellectual foundation of tauopathy research has progressively evolved from defining pathological features to elucidating disease mechanisms and facilitating biomarker development and therapeutic innovation. This evolution is consistent with the temporal changes in research priorities identified by the keyword analysis presented in the following section.

### 4.4 Emerging Research Hotspots

#### 4.4.1 A*β*‐Tau Interaction

Keyword co‐occurrence and temporal evolution analyses identified Aβ and tau as persistent high‐frequency terms, indicating that their interaction remains a central research focus in tauopathy. The sustained prominence of these keywords reflects a growing recognition that Aβ and tau act synergistically rather than independently during neurodegeneration.

Recent studies have demonstrated that Aβ promotes tau hyperphosphorylation, aggregation, and neuronal dysfunction, whereas tau mediates many of the downstream neurotoxic effects induced by Aβ (Medina and Avila 2010; Shafiei et al. 2017; Gamblin et al. 2003; Bloom 2014; Zhang et al. 2021). This evolving understanding has shifted research from investigating individual pathological pathways toward elucidating the molecular interplay between Aβ and tau. Such a transition is consistent with the bibliometric findings, which show sustained attention to both pathological proteins throughout the study period.

The continued prominence of the Aβ‐tau axis also highlights its translational significance. Increasing efforts are being directed toward therapeutic strategies targeting both pathological processes simultaneously, suggesting that combination approaches may represent an important direction for future disease‐modifying therapies in tauopathies.

#### 4.4.2 Neuroinflammation and Inflammasome Activation

Keyword analysis identified neuroinflammation as one of the most prominent emerging research themes in tauopathy, reflecting a growing shift from protein‐centered mechanisms toward the investigation of immune‐mediated neurodegeneration. The increasing frequency of inflammation‐related keywords suggests that neuroinflammatory processes are now recognized as active contributors to tau pathology rather than merely secondary consequences of neurodegeneration.

Recent studies have demonstrated that activation of the inflammasome, particularly the NLRP3 inflammasome, promotes tau hyperphosphorylation, aggregation, and neuronal injury, thereby accelerating disease progression (Ising et al. 2019). Conversely, suppression of inflammasome signaling has been shown to attenuate tau pathology and improve neurodegeneration in experimental models (Stancu et al. 2019, 2022). In addition, accumulating evidence indicates that pathological tau itself can activate microglia and amplify inflammatory responses, forming a self‐perpetuating cycle between tau pathology and neuroinflammation (Panda et al. 2021). These findings are consistent with the increasing prominence of neuroinflammation‐related keywords observed in the present bibliometric analysis.

The emergence of neuroinflammation as a major research hotspot highlights a broader transition from describing pathological protein aggregation to understanding the complex interactions among neurons, glial cells, and the immune system. This evolving perspective has stimulated growing interest in immunomodulatory therapies targeting inflammatory pathways, suggesting that neuroinflammation will remain an important direction for future mechanistic studies and therapeutic development in tauopathies.

#### 4.4.3 Glial Tau Pathology

Keyword evolution analysis indicates that glial cell‐related research has gained increasing attention in recent years, reflecting a growing recognition that tauopathy is a multicellular disorder rather than a purely neuronal disease. This shift has been driven by advances in neuropathology and molecular biology, which have revealed the critical roles of astrocytes and oligodendrocytes in tau propagation, neuroinflammation, and disease progression.

Recent studies have demonstrated that characteristic glial tau inclusions, including tufted astrocytes and coiled bodies, are pathological hallmarks of primary tauopathies such as PSP and CBD (Ikeda et al. 2018; Irwin 2016). Furthermore, accumulating evidence suggests that glial tau pathology may develop independently of neuronal tau pathology, indicating distinct cellular mechanisms underlying different tauopathies (Narasimhan et al. 2020; Spillantini and Goedert 2013). These findings are consistent with the increasing prominence of glial‐related keywords identified in the present bibliometric analysis.

The growing focus on glial biology highlights an important shift toward understanding the complex interactions among neurons, glial cells, and the brain microenvironment. This evolving perspective may facilitate the identification of novel therapeutic targets beyond neurons and contribute to a more comprehensive understanding of tauopathy pathogenesis.

#### 4.4.4 Experimental Models of Tauopathy

Experimental models remain fundamental to elucidating disease mechanisms and evaluating potential therapeutic strategies. The sustained occurrence of model‐related keywords in the present study indicates that improving experimental systems continues to be an important research priority in tauopathy.

Early investigations primarily relied on invertebrate models, whereas transgenic mouse models have become indispensable for reproducing key pathological features, including tau hyperphosphorylation and neurofibrillary tangle formation (Kraemer et al. 2003; Fatouros et al. 2012; Bouleau and Tricoire 2015; Frank et al. 2008; Dujardin et al. 2015). Despite these advances, currently available models remain limited in their ability to fully recapitulate the pathological and clinical heterogeneity of human tauopathies (Sahara and Yanai 2023). Consequently, increasing efforts are being directed toward developing more physiologically relevant and translationally applicable experimental systems.

The continued prominence of model‐related research reflects the growing demand for experimental platforms capable of bridging basic mechanistic discoveries and clinical translation. The development of more representative disease models is therefore expected to accelerate biomarker validation and the evaluation of emerging tau‐targeted therapies.

#### 4.4.5 Tau‐Targeted Therapeutic Strategies

Temporal keyword analysis indicates that tau‐targeted therapy has become one of the fastest‐growing research areas recently, reflecting the increasing recognition of tau pathology as a promising therapeutic target for neurodegenerative diseases. Compared with earlier studies that primarily focused on symptomatic treatment or microtubule stabilization, current research has shifted toward interventions targeting pathological tau species and their propagation.

Among the emerging therapeutic strategies, immunotherapy has received considerable attention, as reflected by the increasing frequency of keywords related to antibodies and immunomodulation. Monoclonal antibodies designed to neutralize extracellular pathological tau have entered clinical evaluation and represent one of the most actively investigated therapeutic approaches (Ji and Sigurdsson 2021). In parallel, strategies aimed at inhibiting tau aggregation, reducing tau phosphorylation, and promoting tau clearance have also attracted growing interest (Limorenko and Lashuel 2022; Wischik et al. 1996; Medina 2018). These developments are consistent with the bibliometric findings, which demonstrate a progressive shift from understanding disease mechanisms to developing disease‐modifying interventions.

Although several tau‐targeted therapies have shown encouraging preclinical results, their clinical efficacy remains to be fully established (Rayman 2023). Nevertheless, the continued emergence of therapy‐related keywords suggests that translational research will remain a major focus of future tauopathy investigations.

#### 4.4.6 Future Perspectives

Beyond identifying current research hotspots, the present bibliometric analysis provides insights into the future direction of tauopathy research. Temporal keyword evolution suggests that research is gradually shifting from descriptive neuropathology toward integrated investigations of molecular mechanisms, biomarker development, and targeted therapeutic strategies. In particular, the growing prominence of neuroinflammation, glial biology, fluid biomarkers, and tau PET imaging indicates an increasing emphasis on multidisciplinary approaches to disease diagnosis and treatment.

Despite the rapid expansion of the field, several challenges remain. The pathological heterogeneity of different tauopathies, the limited translational value of existing experimental models, and the modest clinical efficacy of current therapeutic candidates continue to hinder the development of effective disease‐modifying interventions. Addressing these challenges will require stronger integration of basic research, biomarker discovery, and clinical investigation.

Overall, the bibliometric findings suggest that future research will increasingly focus on elucidating the complex interactions among tau pathology, neuroinflammation, and glial dysfunction while accelerating the translation of mechanistic discoveries into precision diagnostic tools and targeted therapies. These emerging directions are expected to shape the next stage of tauopathy research.

#### 4.4.7 Strengths and Limitations

This study provides a comprehensive and up‐to‐date bibliometric analysis of global tauopathy research from 2003 to 2025 by integrating multiple bibliometric approaches and visualization tools. Compared with previous bibliometric studies, the present analysis incorporated temporal keyword evolution analysis to systematically characterize publication trends, collaboration networks, intellectual foundations, and emerging research hotspots.

Several limitations should be acknowledged. First, the literature search was restricted to the WoSCC. Although WoSCC is widely regarded as one of the most authoritative databases for bibliometric research because of its standardized citation structure and compatibility with bibliometric software, publications indexed exclusively in databases such as Scopus or PubMed were not included, potentially introducing database selection bias. Second, although the search strategy was designed to maximize the coverage of tauopathy‐related literature, relevant publications using alternative terminology or indexing may not have been retrieved. Third, only English‐language publications were included, which may have resulted in language bias and the exclusion of relevant studies published in other languages. Fourth, citation‐based indicators are inherently influenced by publication age, journal visibility, and citation practices. Consequently, recently published studies may have had insufficient time to accumulate citations, leading to an underestimation of their academic impact despite their potential scientific importance. Finally, while keyword co‐occurrence, co‐citation, and network visualization analyses effectively reveal the overall knowledge structure and research evolution, they may not fully capture the complex conceptual relationships within the field.

## 5 Conclusion

This study provides a comprehensive bibliometric analysis of global tauopathy research from 2003 to 2025, offering a systematic overview of publication trends, collaboration networks, intellectual foundations, and evolving research hotspots. The findings demonstrate that tauopathy research has experienced sustained growth over the past two decades, with the United States remaining the leading contributor and several influential investigators establishing the intellectual foundation of the field.

Beyond describing the research landscape, this study highlights the dynamic evolution of scientific priorities. Temporal keyword analysis revealed a gradual transition from classical neuropathological investigations toward multidisciplinary research focusing on neuroinflammation, glial biology, biomarkers, experimental models, and tau‐targeted therapeutic strategies. These findings reflect the increasing emphasis on translational research aimed at improving disease diagnosis and developing disease‐modifying therapies.

By integrating multiple bibliometric approaches, this study provides a comprehensive framework for understanding the historical development, current research priorities, and future directions of tauopathy research. The findings may help researchers identify emerging topics, promote international collaboration, and guide future investigations into the mechanisms, diagnosis, and treatment of tau‐related neurodegenerative disorders.

## Author Contributions

Si‐Yao Zhang: Writing – original draft, conceptualization. Hui‐Jie Ren: Writing – original draft, methodology. Yong‐Jun Peng: Writing – review and editing. Hao‐Yu Liu: software, data curation. Shu‐Ying Xu: Writing – review and editing.

## Funding

The authors have nothing to report.

## Ethics Statement

Ethical approval was not required for this study because all data were obtained from publicly available databases, and no human participants or animals were involved.

## Conflicts of Interest

The authors declare no conflicts of interest.

## Data Availability

The original contributions presented in the study are included in the article/supplementary material, and further inquiries are available by contacting the corresponding/first authors.
