# Correction to “Emerging Trends and Hot Spots in Tauopathy Research (2003–2025): A Bibliometric Analysis”

**DOI:** 10.1002/brb3.71778

**Published:** 2026-09-17

Zhang, S.‐Y., H.‐J. Ren, H.‐Y. Liu, S.‐Y. Xu, and Y.‐J. Peng. 2026. “Emerging Trends and Hot Spots in Tauopathy Research (2003–2025): A Bibliometric Analysis.” *Brain and Behavior* 16: e71645. https://doi.org/10.1002/brb3.71645.

The Funding statement was incorrectly added as “The authors have nothing to report.”

The correct Funding statement should read:

“This work was supported by the Suzhou Municipal Key TCM Specialty (Department of Preventive Treatment of Disease; grant/award number: 2026ZDZK12) and the Natural Science Foundation Project of Nanjing University of Chinese Medicine (grant/award number: XZR2024199).”

We apologize for this error.

